# A new method for multi-site cardiac pacing using leadless electrodes and high frequency transthoracic currents

**DOI:** 10.1038/s41598-025-00692-1

**Published:** 2025-05-21

**Authors:** Rafael Beyar, Rachel Schatzberger, Wisam Darawsha, Rona Shofti, Amir Dagan, Yoram Palti

**Affiliations:** 1 Rambam Health Care Campus and Technion, Israel Institute of Technology, Haifa, Israel; 2 CardoAid, Haifa, Israel; 3 Rappaport Faculty of Medicine, Technion, Israel Institute of Technology, Haifa, Israel

**Keywords:** Physiology, Cardiology

## Abstract

Cardiac pacing using long leads has inherent complications of adhesions, fractures and infection. Current leadless pacemakers include battery-operated devices that are relatively large in size. We present here the initial design and animal experiments with a new ultrathin leadless device, activated by subcutaneous application of short trains of high frequency (250 kHz) alternating electrical fields (HFAF). The myocardial 40 × 0.5 mm rectifying device, implanted in the right ventricle and in epicardial veins, converts the HFAF sinusoidal transients to unidirectional current pulses which stimulate the myocardium. The method was tested in a swine model under open and closed chest conditions for both endocardial and cardiac venous approaches. We demonstrated pacing under all these conditions and measured the thresholds of current, voltage and pulse duration. This method allows for leadless stimulation at multiple points. It may be applied to various pacing needs and has an interesting appeal for CRT Therapy, where multiple electrodes can be implanted, overcoming the limitations of current long pacing leads.

## Introduction

Cardiac pacemakers, including cardiac resynchronization therapy (CRT) involve long leads originating at the implanted stimulator, traversing valves and vascular structures to reach the target cardiac stimulation site. Current lead-dependent technologies are associated with an estimated 15% three-year complication rate, including lead dislodgement and failure^1^, pocket hematoma and infection^2^, all of which contribute to morbidity, mortality, and significant healthcare costs^3^.

Recently, leadless technology that includes the implantation of an entire pacemaker with its battery and electronics at the stimulation site was introduced^4^. Yet, current iterations of leadless pacemakers are limited to one site, and lack the versatility offered by transvenous devices. Leadless stimulation to the LV endocardium as part of CRT therapy using ultrasound energy and an LV endocardial receiver and stimulator is currently under evaluation^5^. The disadvantages of the current leadless devices include size that prevents implantation through the coronary sinus and cardiac venous system, battery depletion and a high risk procedure when extraction is required^6^.

The current paper describes a new method (developed by CardoAid™) for leadless cardiac stimulation using an ultra-thin wire-like device that can be applied to the right ventricle endocardium for direct myocardial stimulation as well as transvenous epicardial stimulation through the coronary sinus. The stimulation current source is a high frequency alternating electric field (HFAF) transients generated in the torso by external or subcutaneous electrodes. Using an implanted ultrathin rectifying device, the captured high frequency current is rectified and stimulates the myocardium at the point of contact. This method can be applied to the needs of multisite cardiac stimulation protocols for CRT as well as other stimulation needs.

## Method of stimulation

The present method is based on the leadless stimulation method first described by Palti in 1962^7,8^. At the basis of this methodology is the elimination of the conducting leads that deliver the stimulating current pulses (0.5–10ms) from the source (usually an external waveform generator) to the stimulating electrodes. Instead, the currents are derived from the high frequency alternating electric fields HFAF generated in the body volume by external or subcutaneous electrodes. Such alternating fields or currents do not stimulate excitable tissues. The reason for the lack of stimulating power is the rapid change in current polarity from one that stimulates to one that inhibits excitation^10^. The kinetics of the electric excitability parameters of the cells are too slow to follow the rapidly alternating field changes. The safety and lack of any stimulating effect of such fields when applied to the human torso have been demonstrated by Blatt et al.^11^.

**Fig. 1:**
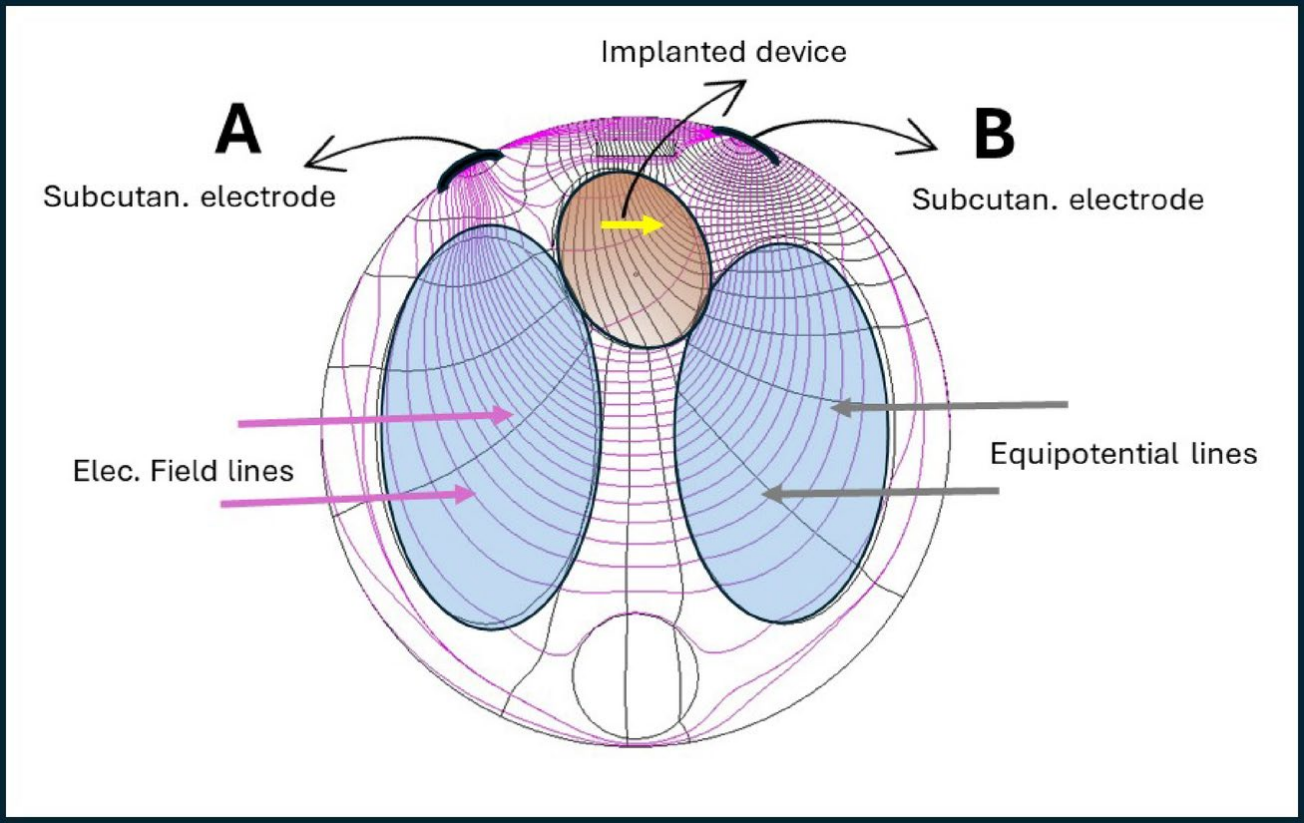
A scheme of the equipotential lines in the torso (sagittal plane) with the heart and lungs shown induced by subcutaneous electrodes A and B. The implanted rectifying device (yellow arrow) is traversing several equipotential lines as shown in the figure.

Electrodes of the rectifying device [RD] positioned at the stimulation site, pick up the fields, rectify the currents they generate and deliver rectified high frequency alternating currents to the selected site. The rectified current pulses have the capability to stimulate and thus pace, provided their distribution is such that they exceed threshold at the site. Figure 1 is a schematic example of the field distribution in a human torso generated by electrodes A and B.

**Fig. 2:**
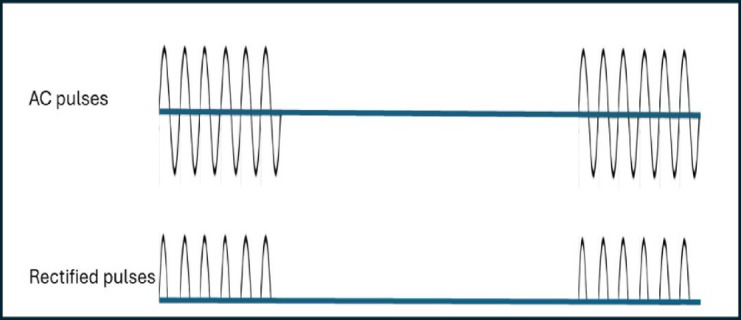
Current pulses consisting of a sine wave (upper trace) & half-rectified current waves (lower trace).

When an implanted RD is placed within the HFAF field in direct contact with the heart muscle, the potential changes are of one polarity only, and the excitable tissues are stimulated by the corresponding induced currents. Figure 2 shows the shape of the pulses of HFAF field in the torso and the rectified currents that do stimulate nerves and muscles at the selected site and thus can pace the myocardium.

**Fig. 3:**
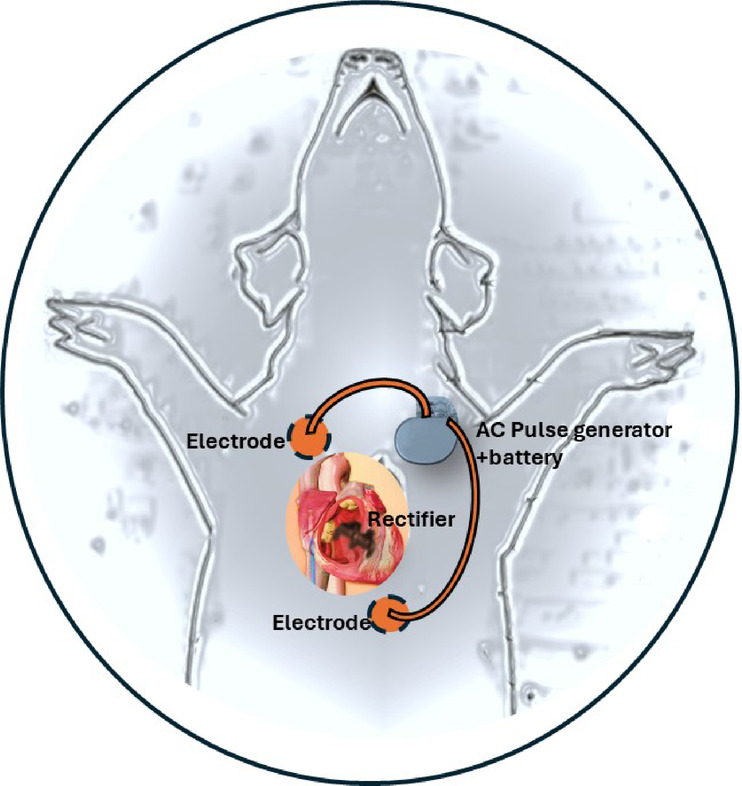
Pacing set-up: Current generator and controller (Implanted under the pectoralis muscle); External subcutaneous electrodes and the implanted rectifying device.

The full setup of the system for cardiac pacing is schematically shown in Fig. 3. It includes a high frequency alternating current generator & controller, HFAF generating electrodes, and the Implanted RD.

In our experiments, the high frequency alternating sine wave generator generates a 250 kHz voltage waveform pulses of 0.1–10 ms and pulse repetition rate equal to the required pacing rate.

### The implanted rectifying device

**Fig. 4:**
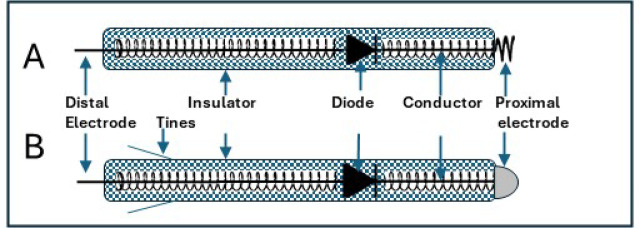
**A** A rectifying device for screw-in Right Ventricle endocardium. **B** A rectifying device for the Left Ventricular epicardial vein.

The implanted RDs consist of a rectifier and a pair of leads. Rectifiers are solid state elements that can be less than 100 microns in size. The rectifiers are connected to a pair of conductors, that are insulated except for their proximal and distal segments. The cathode tip is in the shape of a helical screw (Fig. 4-A) or a dome-like head (Fig. 4-B), that are in direct contact with the myocardium. The RD studied was 40 mm in length and 0.5 mm in diameter. The symmetric alternating polarity current pulses that flow in the torso (Fig. 2) become unipolar (half-rectified) in presence of the device and can thus stimulate the tissue and pace the heart. Threshold stimulating current amplitude ranges were 0.5–10 mA, same as for the conventional pulses used in pacing^12^. We used pacing pulse durations between 0.5-10ms at a pacing rate ranging between 100 and 120 pulse per minute. Note that the half-rectified current pulses (Fig. 2) thus formed have about 50% stimulating power compared with a square pulse. To generate such a current the potential differences between the rectifier contact points are in the order of 1–10 volts.

The implantation of the rectifying device was performed using one of the following methods:

- Epicardial- insertion of the electrode tip directly into the epicardium in open chest swine model.
- RV endocardial- insertion of the screw-in device (Fig. 4-A) through 8 F coronary guiding catheters.
- LV venous epicardial- transcatheter Insertion of the vein device (Fig. 4-B) through the coronary sinus into the selected epicardial vein over the LV lateral wall. The current is injected into the excitable myocardium through the thin vein wall.

### Animal experiments: methods

A total of sixteen healthy male pigs (13 in a closed chest study), 55–65 kg in weight were studied. The pigs (Landrace farm animals) were purchased by the Pre-clinical research authority of the Technion from an approved vendor, the Technion facility is an AAALA (Association for Assessment and Accreditation of Laboratory Animal Care) accredited facility.

The pigs were anesthetized with a mixture of Ketamine (20 mg/kg) + ACP 1 mg/kg IM(or xylazine1mg/kg) followed by intravenous administration of propofol (5–7 mg/kg) and Fentanyl (3–10 µcg/kg/hr) through an ear vein access, intubated, and ventilated. Anesthesia was maintained with inhalation of isoflurane (1.5–2%) in 100% oxygen. Electrocardiogram leads were attached to the limbs, and the left femoral artery was cannulated (5 F sheath) for monitoring blood pressure (BP); A transonic^®^ Flowmeter T106 probe was positioned over the carotid artery to measure blood flow.

The study was approved by the IACUC (Institutional Animal Care and Use Committee) of the Technion, Israel Institute of Technology. All procedures complied with the Animal Welfare Act of 1966 (P.L. 89–544), as amended by the Animal Welfare Act of 1970 (P.L.91–579) and 1976 (P.L. 94–279) and reported in accordance with ARRIVE guidelines.

### Open chest direct epicardial stimulation

3 pigs were studied. After inducing anesthesia as above, local anesthesia was induced, median sternotomy was performed, and the pericardium was opened to expose the heart. The cathode (Proximal electrode) of the rectifying device was attached directly to the epicardium over the LV wall or pierced into the muscle, while the anode edge was attached to the muscles of the chest wall. The external electrodes were tunneled subcutaneously over the chest.

### Close chest RV and LV epicardial vein stimulation

After inducing anesthesia as above, the jugular vein was accessed and a 9 F introducer sheet was inserted. Under X-ray fluoroscopy (Ziehm Vision), an 8 F multipurpose catheter was guided through the jugular vein to the vena cava, right atrium and to the right ventricle (RV). A ventricular rectifying pacing device (Fig. 4-A) was delivered through the catheter, anchored into the RV septum by screwing and disconnected from the catheter by a push mechanism.

**Fig. 5:**
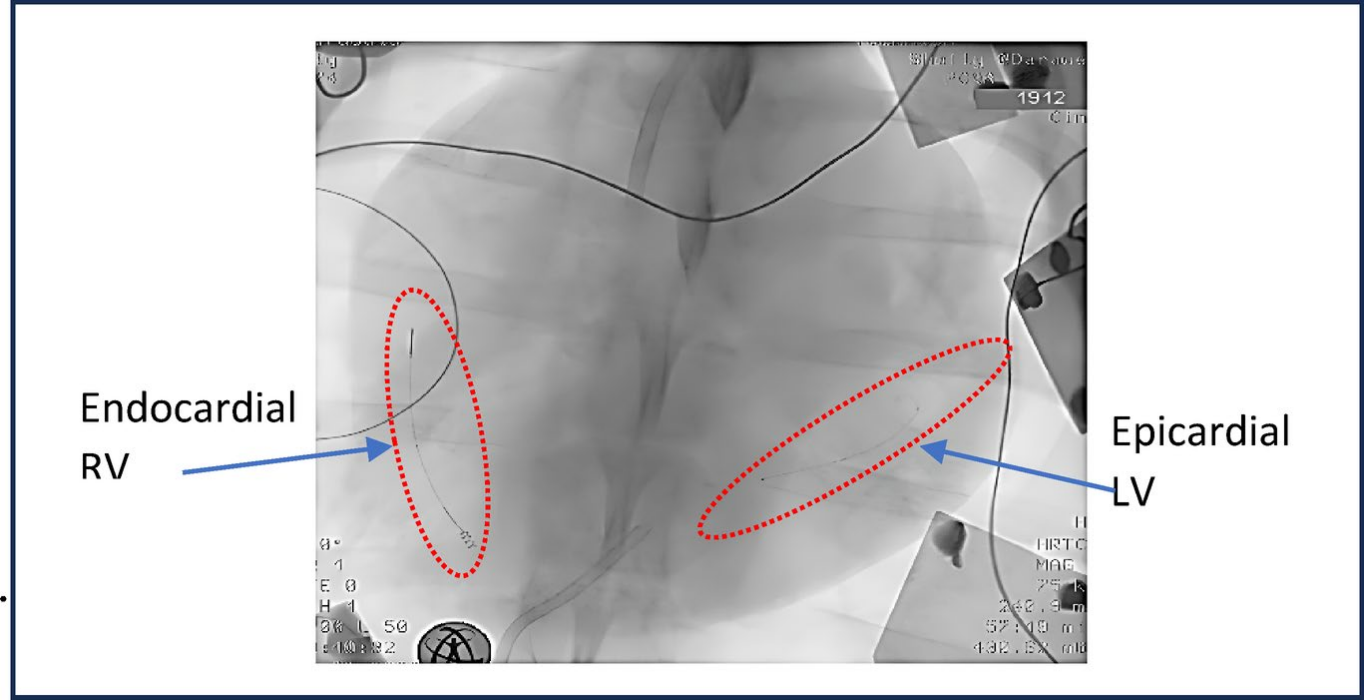
X-ray imaging after implanting endocardial RV and epicardial LV rectifier electrodes.

The epicardial vein rectifying pacing devices (Fig. 4-B) were introduced through the coronary sinus (CS) into a vein on the lateral wall of the LV (Fig. 5). This device was essentially similar to the one implanted in the RV but featured a dome-shaped head at the cathode end, in contact with the internal venous wall. To prevent backward sliding 2 tines were added to the device. The direction of the implanted RD was determined by fluoroscopy (Fig. 5).

Subcutaneous electrodes (Cu, 5 × 5 cm) were implanted on both sides of the torso. The electrodes generating the HFAF were aligned with the direction of the implanted RD as seen by fluoroscopy. These electrodes were connected to the external pulse generator which delivered 250 kHz AC stimulation pulses of 0.5-10ms duration, at a predetermined pacing frequency. The same electrodes were used to capture the resulting ECG signals and determine the pacing rate. In order to override the natural heart rate of the animal, the pacing frequency was set at about 20% above the prevailing heart rate.

The variability of the direction of the electrical field with respect to the direction of the electrodes was tested by varying the location of the subcutaneous electrodes.

### Impedance measurement

The tissue and Electrode-tissue contact impedance determine the voltage required for generating super-threshold stimulating currents. The impedance was measured by adding a 50ohm resistor (small relative to the measured impedance) in series with the proximal RD. The potential drop along the resistor served to calculate the current injected into the tissue. From this current value and the potential drop across the tissue junction, the impedance was calculated to be in the range of 280–330 ohm.

## Results

Following implantation of the rectifying device, no significant changes in ECG and blood pressure were detected. The results of the different experimental models are detailed in Table 1. As shown, different combinations of external voltage amplitudes and pulse durations were tested, and the stimulation thresholds were determined. The external field parameters used in these studies were: AC frequency − 250 kHz, AC pulse duration of 0.5–10ms and AC external pulse current amplitude of 2.5 Amp (peak to peak) driven by 60–170 Volts p-p.

**Fig. 6:**
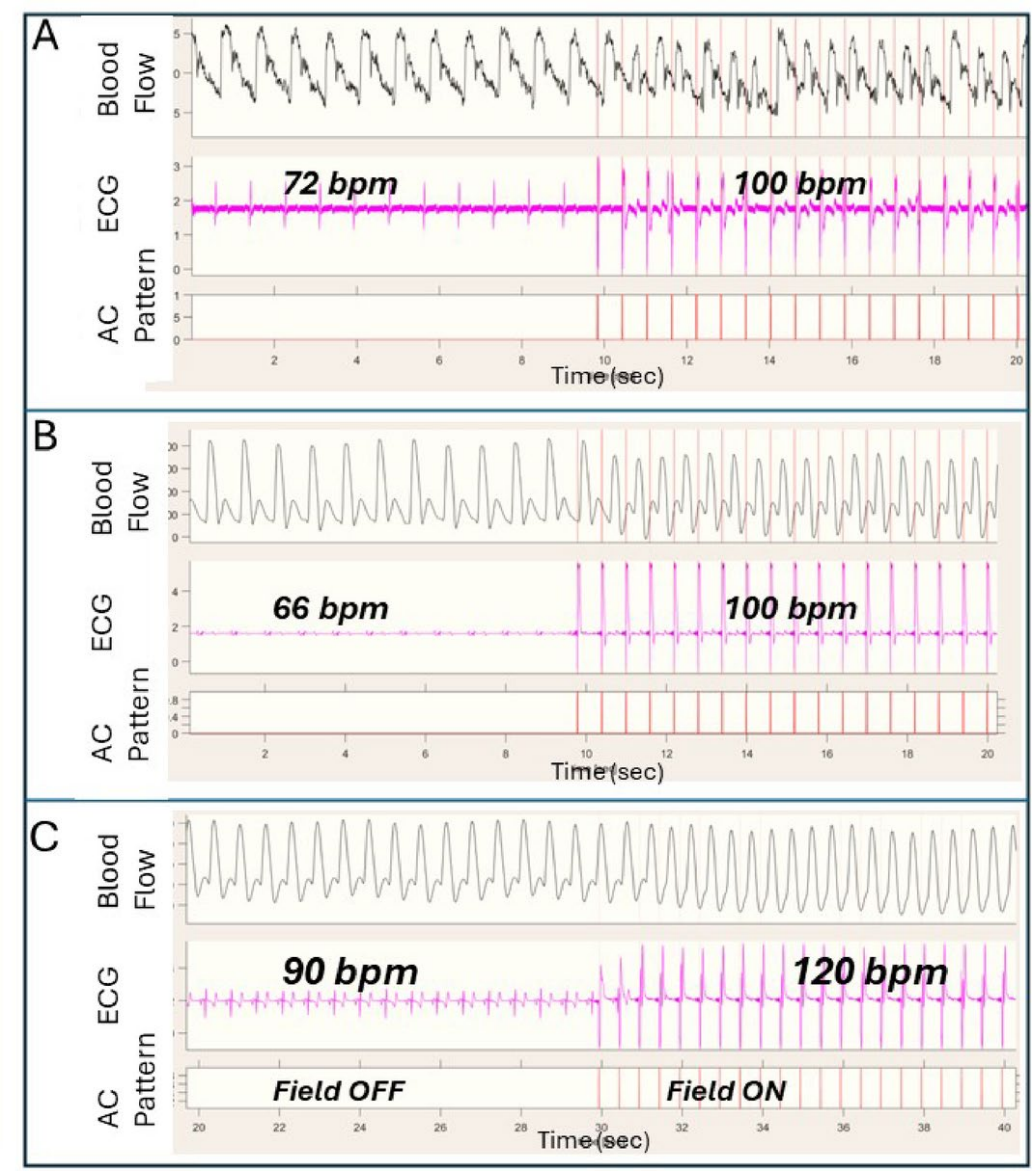
Data recorded while pacing: (**A**) open chest epicardial rectifying device; (**B**) RV endocardial rectifying device; (**C**) LV epicardial vein rectifying device. In each set, the blood flow recorded from the carotid artery (FLOW), the ECG and the pacing high frequency AC voltage trace are shown.

Figure 6 shows the pacing results in 3 examples from the direct epicardial (Fig. 6-A), the RV endocardial (Fig. 6-B) and the LV epicardial vein (Fig. 6-C) approaches.

As presented in Table 1, in 6 animals wireless capture of RV pacing was demonstrated, and in 3 animals LV wall stimulation through the epicardial veins was demonstrated.

The stimulating field parameters of the RV and the LV epicardial parameters were of similar ranges.

In another set of experiments, we measured the current threshold for stimulation. The threshold was 2.5 mA (1ms) like the reported current threshold for standard cardiac pacing^12^.

**Table 1:** Implantation studies and stimulus parameters.

Type of Study	Implantation site	Notes	Stimulus parameters		
			AC amplitude (Volt)	Current in torso (Amp)	Pulse duration (ms)
Open chest	LV epicardial #1	Prime style device model. The field electrodes were attached to the inner chest wall	60–170	1.2–2.5	1–5
	LV epicardial #2	Checking different types of rectifiers for stimulation current threshold. AC was applied directly on device		* Threshold current 2–5 mA	1–10
	LV epicardial #3	Different types of rectifiers attached externally to LV wall	140	1.6	10
Closed Chest	RV	Implant in septum wall near the apex	116–150		1–10
			148–168	2.5–3.3	5–10
			40–60	0.85–1.5	1–10
			140	3	10
			120	2	0.5-1
			170	3	1
	LV	LV epicardial vein through the CS	160	2.5	10
			70–85	1.5-2	1–10
			160	3	1

As can be seen in Fig. 6 the ECG signals and the blood flow signals respond immediately to the HFAF rate and show steady pacing. Note that the depicted ECG signals show the artefact of the high frequency electric field pulses.

Most of the devices were anchored in the mid-RV ventricular septum. The actual potential difference induced by the field across the device is a cosine function of the angle between the external field direction and the actual device axis. It was investigated by changing the direction of the field by selecting different locations for the paired subcutaneous electrodes generating the field. As expected, from the cosine relationship, a change of about +/-30 degrees did not significantly affect the excitation threshold.

**Fig. 7:**
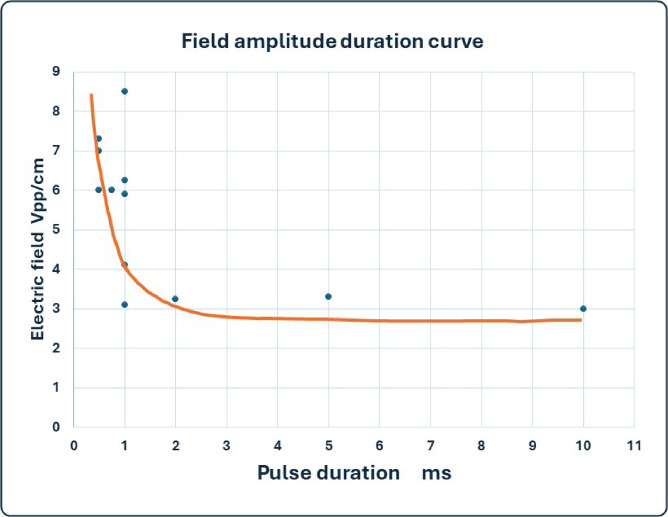
The electric field amplitude (Volts/cm) vs. pacing pulse duration (in ms).

In Fig. 7 we show the typical field amplitude duration curve. Note that typically 1ms pulses are used in cardiac pacing.

## Discussion

We have described a new method of myocardial stimulation based on applying short waveform trains of high frequency alternating electrical field (250 kHz) to the torso. Implanted RDs induce myocardial stimulation by converting the AC pulses to unidirectional half-rectified voltage and current (Fig. 2), injected into the myocardium. We have shown the feasibility of that pacing method for epicardial approach, direct RV endocardial approach and coronary sinus transvenous approach. The method may be applied to CRT for heart failure patients, using one or more rectifier devices implanted through the coronary sinus and epicardial venous system. This method uses miniature rectifiers, without long connecting leads, as direct stimulators to the myocardium. We have applied the method in an acute swine model, have shown the feasibility for stimulating the myocardium and have presented the voltage and current parameters that are needed.

The current lead-based pacemakers and CRT systems offer a good solution for the treatment of brady-arrhythmias as well as other pathologies, however they are associated with a relatively high risk of complication in the early and especially in the long-term follow up that range between 10 and 15% in 5 years after implantation^13^. The complications include risk of endovascular infection that is in strict relation to the number of leads implanted and to their length across the vascular system, lead failure that includes insulation breach and lead fracture, sensing and threshold problems and vascular complications like fibrosis and vein occlusion in different sites along the lead course.

The advantage of the present pacing method is the ability to pace from different sites, such as the right ventricle endocardium and the left ventricle epicardium, either by a pericardial approach or through the coronary veins. The system is capable of simultaneous pacing from different venous sites through the coronary sinus. Additionally, the rectifying devices can be implanted in various locations in the heart and can be used for direct left bundle branch pacing and lead to physiological conduction system pacing. Obviously, the small myocardial rectifying devices, by not traversing the venous system, reduce the risk of lead infection. In case of need for extraction, these 4 cm long devices may be caught by available snare devices and retracted. However, we have not conducted yet long term studies that will address these and other issues. Note that it is very likely that results similar to the above can be obtained using 1–2 cm long rectifying devices and higher amplitude fields.

The safety of using high frequency alternating fields across the chest has been demonstrated by the Novocure therapy for lung cancer^11^. In that case the electrical fields were applied to the chest for long periods (week to months) and were shown to be free of any stimulatory or adverse events in the body. In the current method the high frequency field is applied in short pulses at the required pacing rate, and therefore the electrical field used is limited by the duration of the stimulation. When compared to other leadless pacemakers (Table 2), it is clear that the current RD is of a much smaller diameter than that used today. This advantage is of appealing application to CRT, where multiple electrodes may be implanted. It can also be used in LV endocardial approach, as was used by Jashan et al.^14^. The ability to simultaneously activate several LV wall electrodes is also of interest and requires further studies. Another potential application that has not been tested is pacing of the left bundle for better synchronization.

**Table 2:** Leadless pacemakers’ sizes. (Taken from Jashan et al.^14^).

	Nanostim St Jude	Micra Medtronic	Empower Boston Scientific	Wise-CRT EBR systems	Rectifier CardoAid
Diameter (mm)	5.99	6.7	6	2.7	0.5
Length (mm)	41.4	25.9	31.9	9.1	40
Location	RV + RA	RV + RA	RV + RA	LV	RV, LV epi, LV Venous

Biphasic stimulating currents may have an advantage in electrode contact preservation during long-term activity and in providing better performance, based on a study by Mower et al^15^. While we can produce only monophasic pulses using rectified HFAF, we have clearly shown the feasibility of using this as a pacing method. It is to be emphasized that except for the paper cited above, there is very little data comparing monophasic with biphasic waveforms, and most pacemakers do not publish their pacing pulses (Mono- or biphasic forms) in their research articles. Further studies will have to validate the long-term efficiency of our pacing method as compared to current methods.

Obviously, in this publication we were limited to acute experiments. The limitations of the method that need to be addressed in the future include the long-term durability of the stimulation method. As we know from the classical pacing methods, the threshold for stimulation may increase in time due to fibrosis and may require adjustment of the voltage and duration of the stimulation pulse. In our case this will have to be studied in chronic models and in patients.

In summary, we have shown the feasibility of a novel pacing method using high frequency external fields for stimulation by implanted rectifying devices in direct contact with the myocardium. This may have direct application to CRT for heart failure patients and other pacing indications.

## Data Availability

The datasets recorded and analyzed during the current study are available from the corresponding author on reasonable request.

## References

[1] Qin, D. et al. Short- and Long-Term risk of lead dislodgement events: Real-World experience from product surveillance registry. *Circul. Arrhythmia Electrophysiol.* **15** (8), e011029. 10.1161/CIRCEP.122.011029 (2002). 10.1161/CIRCEP.122.011029 35925831

[2] Datta, G. A study on pacemaker pocket infection. *J. Cardiol. Cardiovasc. Med.* **5**, 056–059. 10.29328/journal.jccm.1001087 (2020).

[3] Aatish Garg, Jayanthi, N. et al. Morbidity and mortality in patients precluded for transvenous pacemaker implantation: Experience with a leadless pacemaker. *Heart Rhythm*. **17** (12), 2056–2063. 10.1016/j.hrthm.2020.07.035 (2020). 32763431 10.1016/j.hrthm.2020.07.035

[4] Vouliotis, A. I., Roberts, P. R., Dilaveris, P. & Gatzoulis, K. Arthur Yue, and Konstantinos Tsioufis. Leadless pacemakers: Current achievements and future perspectives. *Eur. Cardiol. Rev.* **18**, e49 (2023). 10.15420/ecr.2022.32 PMC10466270 37655133

[5] Majd, E. et al. First-in-human wireless left ventricular endocardial pacing in a patient with obliterated venous system and complete heart block. *Heart Rhythm Case Rep.* **8**, 497–450 (2022). 10.1016/j.hrcr.2022.04.010 PMC9289050 35860785

[6] Pearce, P. JA, BourlandJD, Neilsen, W., GeddesLA & Voelz, M. Myocardial stimulation with ultrashort duration current pulses. *Pace* **52**, 58 (1982). 10.1111/j.1540-8159.1982.tb02191.x 6181474

[7] Palti, Y. Stimulation of muscles and nerves by means of externally applied electrodes. *Bull. Res. Counc. Isr. Sect. E Exp. Med.* **10**, 54–56 (1962). 14038165

[8] Palti, Y. Stimulation of internal organs by means of externally applied electrodes. *J. Appl. Physiol.* **21**, 1619–1623 (1966). 5923236 10.1152/jappl.1966.21.5.1619

[9] Kirson, E. D. et al. Alternating electric fields arrest cell proliferation in animal tumor models and human brain tumors. *Proc. Natl. Acad. Sci.*, 104 (issue 24), 10152–10157 (2007). 10.1073/pnas.0702916104 10.1073/pnas.0702916104 PMC1886002 17551011

[10] Weinberg, S. H. High-Frequency stimulation of excitable cells and networks. *PLoS ONE*. **8** (11), e81402. 10.1371/journal.pone.0081402 (2013). 24278435 10.1371/journal.pone.0081402 PMC3835437

[11] Blatt, R. et al. Vivo safety of tumor treating fields (TTFields) applied to the torso. *Front. Oncol.* **11**, 670809. 10.3389/fonc.2021.670809 (2021). 34249709 10.3389/fonc.2021.670809 PMC8264759

[12] Issa, Z. F., Miller, J. M. & Zipes, D. P. Clinical arrhythmology and electrophysiology (A companion to Braunwald’s heart Disease). *Electrophysiological Test. Chapter*. **4**, 62–91. 10.1016/b978-1-4557-1274-8.00004-x (2012).

[13] Brignole, M. et al. 2013 ESC guidelines on cardiac pacing and cardiac resynchronization therapy. *Europace* **15**, 1070–1118. 10.1093/europace/eut206 (2013). 23801827 10.1093/europace/eut206

[14] Jashan & Gill Emerging technologies in electrophysiology: From Single-Chamber to biventricular leadless pacemakers. *Cardiology* **147**, 179–190. 10.1159/000521976 (2022). 35038698 10.1159/000521976

[15] Mower, M. M., Hepp, D. & Hall, R. Comparison of chronic biphasic pacing versus cathodal pacing of the right ventricle on left ventricular function in sheep after myocardial infarction. *Ann. Noninvasive Electrocardiol.* **Apr;16** (2), 111–116 (2011). 21496160 10.1111/j.1542-474X.2011.00430.x PMC6932136

